# A preliminary study of peripheral T‐cell subsets in porokeratosis patients with *MVK* or *MVD* variants

**DOI:** 10.1002/ski2.82

**Published:** 2021-12-16

**Authors:** L. Tao, Y. K. Huang, K. X. Yan, C. H. Li, L. Shen, Z. H. Zhang

**Affiliations:** ^1^ Department of Dermatology Huashan Hospital Shanghai Medical College of Fudan University Shanghai China; ^2^ Department of Dermatology Xiamen Chang Gung Hospital Xiamen China; ^3^ Genesky Biotechnologies Inc Shanghai China; ^4^ Shanghai Institute of Immunology Shanghai Jiao Tong University School of Medicine Shanghai China

## Abstract

**Background:**

Porokeratosis (PK) is considered a skin‐specific autoinflammatory keratinization disease. Intriguingly, four causative genes of PK are in turn arranged in mevalonate pathway, with *MVD* variants being the commonest followed by *MVK* variants in a cohort of Chinese patients. Evidence indicates that mevalonate metabolites induce trained immunity in human monocytes and regulate T cells at multiple levels. Of note, γδT cells are dually regulated by intracellular and extracellular mevalonate metabolism.

**Aims:**

To identify the possible differences in T‐cell between *MVK* or *MVD* variants from PK patients.

**Materials & Methods:**

Targeted exome sequencing and exonic CNV screening were performed in 26 patients with PK. Sanger sequencing was used to validate all identified variants. Among them, 22 patients were identified with *MVK* or *MVD* variants. PBMCs from 22 PK patients and 27 normal controls (NCs) were analysed by flow cytometry for the frequencies of T cells subsets, including IFN‐γ‐, and TNF‐α‐producing T cells.

**Results:**

There were 14 mutations identified in the 26 PK patients, including 6 novel mutations (*MVK*: c.118_226+1337dup, c.388_392delGATATinsC, c.613A>T, c.768G>C, and *MVD*: c.250C>T, c.988T>G). In contrast to NCs, significantly decreased frequencies of CD8^+^ and Vγ9Vδ2 T cells were observed in the PK patients with *MVD* variants. Moreover, it was found that dysregulated secretion of pro‐inflammatory cytokines by T cells in both PK patients with *MVK* and *MVD* variants.

**Conclusions:**

Our findings enriched the Human Gene Mutation Databases and showed probable differences in peripheral T cells subsets between PK patients and controls.

## Graphical abstract

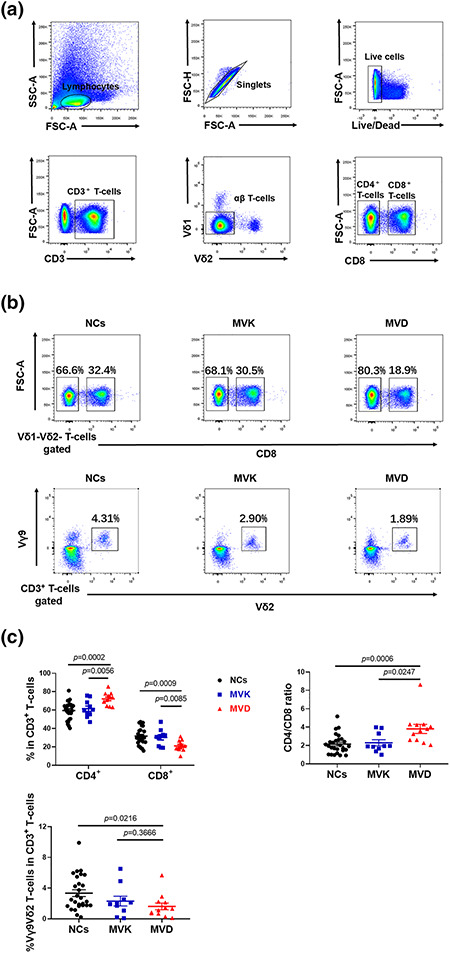

## 1 INTRODUCTION

As a skin‐specific autoinflammatory keratinization diseases (AIKDs), porokeratosis (PK, MIM 175800) is inherited in an autosomal dominant pattern, whose genetic causative factors are associated with the hyperactivation of innate immunity, mainly in the epidermis and upper dermis.^1^ Intriguingly, four causative genes of PK are in turn arranged in mevalonate pathway, that is, mevalonate kinase (*MVK*), phosphomevalonate kinase (*PMVK*), mevalonate (diphospho) decarboxylase (*MVD*) and farnesyl diphosphate synthase (*FDPS*).^2^, ^3^ Among them, *MVD* variants are the commonest cause followed by *MVK* variants in a cohort of Chinese patients.^3^, ^4^ Notably, the patients with *MVK* variants generally showed the widest range of phenotypes in terms of both the number and the size of lesions**.** Giant plaque‐type PK ptychotropica (PPt) appears to be a unique phenotype associated with *MVK* variants. The lesions of *MVD* variants tend to be more homogeneous and superficial than those carrying *MVK* variants. The possible reason is that MVK and MVD deficiencies result in different metabolites of the mevalonate pathway. Generally, it is accepted that mevalonate metabolites regulate T‐cell at multiple levels.^5^ Based on the previous findings, we hypothesized that T‐cell bearing heterozygous mutations in mevalonate pathway might be altered and involved in the autoinflammation of PK. The possible reason is that the different abnormal metabolic intermediates produced by MVK and MVD variants might have different effects on T‐cell activation and cytokine production. In this study, we preliminarily analysed the distribution and cytokine production of peripheral T‐cell subsets in PK patients with *MVK* or *MVD* genotype.

## 2 REPORT

The study was approved by the by the Scientific Ethical Committee of Fudan University, and all participants provided written informed consent. From 2018 to 2019, peripheral blood samples were collected from 26 patients with PK (13 males and 13 females; mean age 53 ± 3 years). They were diagnosed by at least two experienced dermatologists, based on both clinical features and histological examinations. In view of the family history, these patients were divided into 15 familial and 9 sporadic cases. In addition, blood samples were collected from 27 healthy adult individuals (9 males and 18 females: mean age 42 ± 2 years). We determined the genomic variations in the PK patients by targeted exome sequencing and CNV analysis.^3^ As indicated in Table 1, 14 mutations were identified in *MVK*, *PMVK* and *MVD*, including four novel mutations in *MVK* (c.118_226+1337dup, c.388_392delGATATinsC, c.613A>T, c.768G>C) and two novel mutations in *MVD* (c.250C>T, c.988T>G) (Figures S1 and S2). The novel mutations found in this study were confirmed to be absent in both gnomAD Exomes and ExAC East Asian database. The commonest *MVD* mutation (c.746T>C) was identified in seven of the unrelated patients, accounting for 26.9% of 26 PK patients. No mutation was found in three sporadic PK patients.

**TABLE 1:** Characterization of 14 variants identified in 21 of the 26 patients

No.	Gene	Locus reference genomic	Mutation	Exon	Predicted protein alternation	Mutation type	ACMG	CADD	SIFT score	POLY ‐PHEN score	Mutation‐Taster score	ExAc_EAS^a^	GenomeAD_Exomes_EAS^b^	Cases		References
														Familial (affected/ unaffected)	Sporadic	
1	*MVK*	NM_000431.2	c.118_226+1337dup	2,3	p.?	Duplication								F‐2(1/0)		
2	*MVK*	NM_000431.2	c.388_392delGATATinsC	5	p.(Asp130Profs*2)	Frameshift Substitution	Pathogenic							F‐9(1/0)		
3	*MVK*	NM_000431.2	c.451G>A	5	p.(Val151Met)	Missense	Likely pathogenic	5.9687	0.01	0.938	0.999995			F‐11(1/0)		Zhang et al.^3^
4	*MVK*	NM_000431.2	c.613A>T	6	p.(Asn205Tyr)	Missense	Likely pathogenic	6.1694	0	1	1			F‐1(3/0)		
5	*MVK*	NM_000431.2	c.710C>A	8	p.(Thr237Asn)	Missense	Likely pathogenic	4.9806	0	0.992	0.999254			F‐14(1/0)		Zhang et al.^3^
6	*MVK*	NM_000431.2	c.768G>C	8	p.(Lys256Asn)	Missense	Likely pathogenic	5.998	0.005	0.86	1				S‐4	
7	*MVK*	NM_000431.2	c.1039+2T>C	10	p.?	Splice_Site	Pathogenic	4.744			1			F‐8(1/0)		Zhang et al.^2^, ^3^
8	*MVK*	NM_000431.2	c.1126G>A	11	p.(Gly376Ser)	Missense	Likely pathogenic	5.6853	0	0.996	0.999999			F‐15(1/0)		Zhang et al.^2^, ^3^
9	*PMVK*	NM_006556.3	c.412C>T	4	p.(Arg138*)	Nonsense	Pathogenic	13.0376	1	0.735406	1			F‐6(1/0)		Zhang et al.^3^
10	*MVD*	NM_002461.1	c.250C>T	3	p.(Arg84Trp)	Missense	Uncertain significance	3.1932	0.008	0.97	0.987	0.0002	0.0002	F‐7(1/0)		
11	*MVD*	NM_002461.1	c.383C>T	4	p.(Ala128Val)	Missense	Uncertain significance	5.6033	0	0.998	0.999994	0.0002	0.0001		S‐6	Zhang et al.^3^
12	*MVD*	NM_002461.1	c.746T>C	7	p.(Phe249Ser)	Missense	Likely pathogenic	5.2987	0	1	0.999989	0.0005	0.0002	F‐3(1/0); F‐4(1/0); F‐5(1/0); F‐10(1/0); F‐12(1/0); F‐13(1/0)	S‐2	Zhang et al.^3^; Li et al.^4^; Leng et al.^6^; Kubo et al.^7^
13	*MVD*	NM_002461.1	c.988T>G	8	p.(Phe330Val)	Missense	Uncertain significance	−0.0172	0.001	0.009	1				S‐1	
14	*MVD*	NM_002461.1	c.1111_1113del	9	p.(Ile371del)	In_Frame_Del	Uncertain significance					0.0002	0.0001		S‐3; S‐5	Zhang et al.^3^

^a^ Exome Aggregation Consortium_East Asian allele frequency.

^b^ The Genome Aggregation Database_Exomes_ East Asian allele frequency.

**FIGURE 1:**
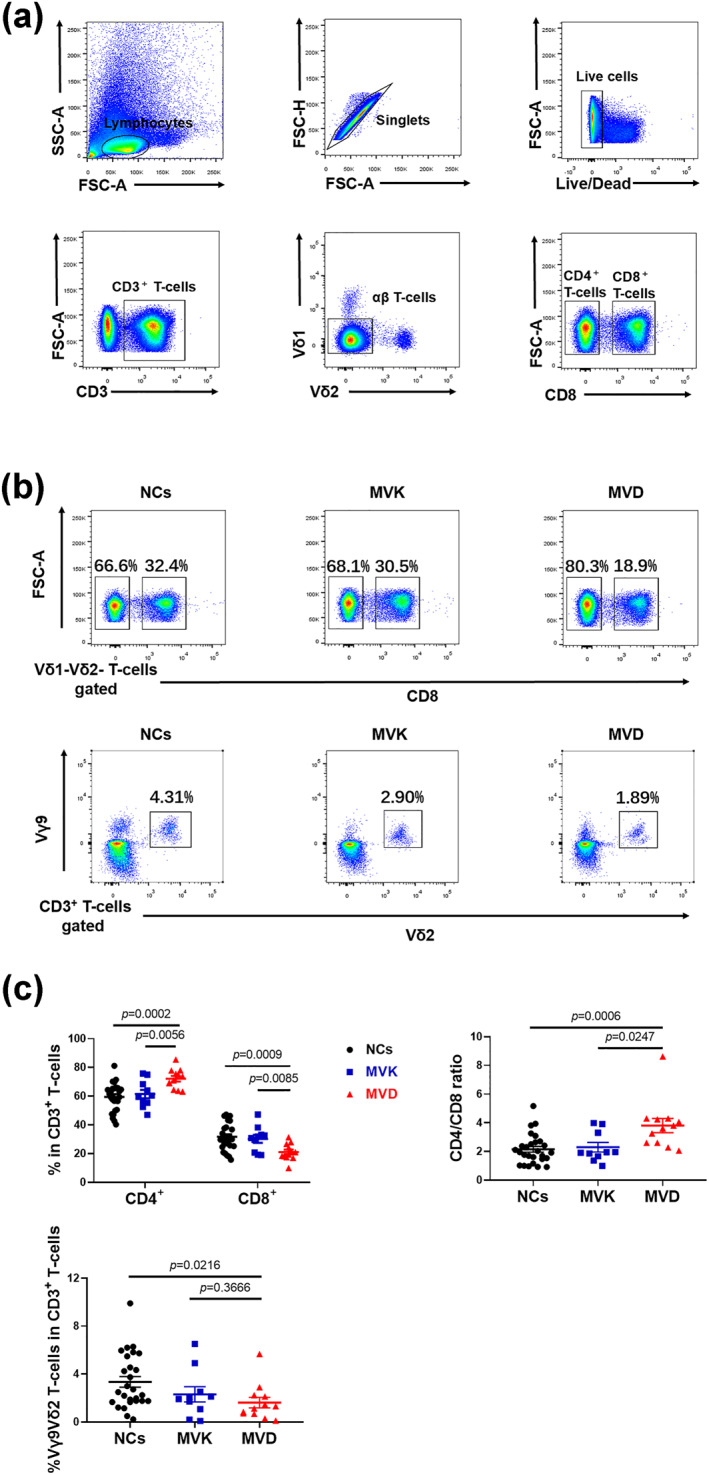
Gating strategy for flow cytometry (a), representative flow cytometry analyses (b), and scatterplot graphs (c) showed the frequencies of CD4+, CD8+ and Vγ9Vδ2T cells in the CD3+ T‐cell subsets and the ratio of CD4^+^ T‐cell/CD8^+^ T‐cell

**FIGURE 2:**
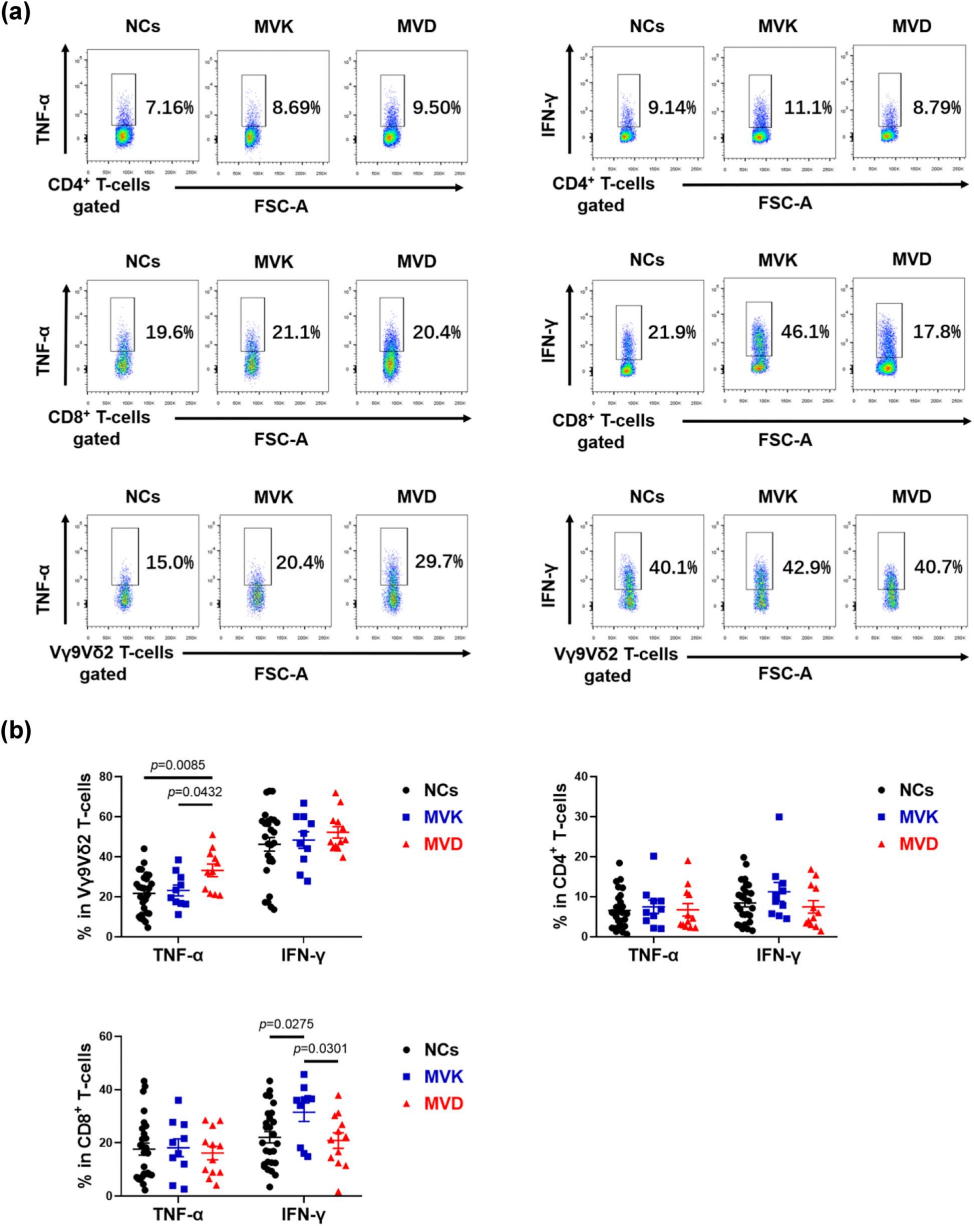
Representative flow cytometry analyses (a) and scatterplot graphs (b) of TNF‐α and IFN‐γ production in CD4+, CD8+ and Vγ9Vδ2T cells in the PK patients with MVK and MVD variants. MVK, the PK patients with *MVK* variants (*n* = 10); MVD, PK patients with *MVD* variants (*n* = 12); NCs, normal controls (*n* = 27)

PBMCs from 22 PK patients with *MVK* or *MVD* variants and 27 normal controls (NCs) were analysed by flow cytometry for the frequencies of T‐cell subsets, including IFN‐γ‐, and TNF‐α‐producing T‐cells. As indicated in Figure 1, the PK patients with *MVD* variants exhibited a significant decrease in the frequencies of peripheral CD8^+^ and Vγ9Vδ2T cell in the CD3^+^ T‐cell subsets compared with that of the NCs (*p* = 0.0009 and *p* = 0.0216, respectively). Therefore, the PK patients with *MVD* variants had a correspondingly higher CD4/CD8 ratio compared to that of the NCs (*p* = 0.0006). In addition, we found that the percentages of total Vδ1^+^, Vδ1^+^Vγ9^+^ and Vδ1^+^Vγ9^−^ T‐cells in the CD3^+^ T‐cell subsets remained unchanged in the PK patients with either *MVK* or *MVD* variants compared with that of the NCs (Figure S3). Figure 2 showed IFN‐γ production by CD8^+^ T‐cell was increased in PK patients with *MVK* variants compared with that of the NCs and PK patients with *MVD* variants (*p* = 0.0275 and *p* = 0.0301, respectively). Moreover, TNF‐α production by Vγ9Vδ2 T‐cell was significantly increased in the PK patients with *MVD* variants compared with that of the NCs and the PK patients with *MVK* variants (*p* = 0.0085 and *p* = 0.0432, respectively).

Different from the patients with *MVK* variants, we observed that there were significant decreases in percentages of both CD8^+^ and Vγ9Vδ2T cells in the CD3^+^ T cells subsets in those with *MVD* variants. A reduction in the number of peripheral γδT cells, along with the elevated numbers in the skin, has been reported in patients with psoriasis.^8^ A possible explanation was that CD8^+^ and Vγ9Vδ2T cells might home to the skin in the PK patients with *MVD* variants. As for non‐conventional lymphocytes, human γδT cell acts as the first line of defence and bridge the innate and adaptive immune systems, representing <5% of peripheral T‐cell in the adult human peripheral blood. γδT cells are at the crossroads of autoinflammation and autoimmunity, which produce cytokines and chemokines to promote the development of mixed‐pattern immune‐mediated inflammatory diseases.^9^ It is well established that multiple phosphorylated mevalonate metabolites are potent agonists of Vγ9Vδ2T cells.^5^ As the major peripheral γδT‐cell subsets, Vγ9Vδ2T cells are dually regulated by intracellular and extracellular mevalonate metabolism. Moreover, the peripheral Vγ9Vδ2T cells from PK patients with *MVD* variants showed a higher proportion expressed TNF‐α compared with NCs. In a different way, the dysregulated secretion of IFN‐γ by CD8^+^ T‐cells were observed in the PK patients with *MVK* variants. It is noteworthy that the IFN‐γ signalling for CD8^+^ T‐cells differentiation are delivered early in the immune response. The autocrine IFN‐γ signalling plays an important role in Th1 differentiation and CD8^+^ T‐cells cross‐priming.^10^, ^11^ It suggested that autoreactive CD8^+^ and γδT cells might play a critical role in the skin‐specific autoinflammatory PK. However, it was not known to what extent, if any, the pro‐inflammatory effects of these T‐cells might affect the pathogenesis of PK.

Danger signals from exogenous pathogens and endogenous keratinocyte death might trigger skin inflammation. Under certain circumstances, genetic defects in mevalonate pathway might block DNA degradation during epidermal cornification and develop a vertical ‘column’ of parakeratosis, histologically defined as a cornoid lamella (CL). In the context of a heterogeneous group of disease, CL unifies all phenotypes of PK. It is remarkable that non‐specific papillary dermal lymphocytic infiltration are frequently seen under the CL.^12^ The local immune cell infiltration and chronic activation are involved in the pathomechanisms of PK.^13^ The limitations of this study include a small sample size and the lack of comparison of CD8^+^T and γδT cells in the lesions of PK with *MVK* or *MVD* variants. Further investigation is necessary to explore resident T‐cells in the lesions of PK.

Taken together, our findings showed alterations in peripheral T‐cell subsets found in PK patients and provided the cues to further studies on autoreactive CD8+T and γδT cells in the pathogenesis of autoinflammatory keratinization.

## CONFLICT OF INTEREST

The authors declared no conflicts of interest.

## AUTHOR CONTRIBUTIONS

**L.** **Tao:** Data curation; Formal analysis; Investigation; Writing – original draft; Writing – review & editing. **K. X.** **Yan:** Resources. **C. H.** **Li:** Formal analysis; Software; Validation. **L.** **Shen:** Conceptualization; Data curation; Formal analysis; Investigation; Supervision. **Z. H.** **Zhang:** Conceptualization; Funding acquisition; Investigation; Supervision; Validation; Writing – original draft; Writing – review & editing.

## Supporting information

Supporting Information S1

## Data Availability

The data that support the findings of this study are openly available at https://doi.org/10.17632/n93jd2znbm.1.
