# Supplementary material for: A preliminary study of peripheral T‐cell subsets in porokeratosis patients with MVK or MVD variants
Source: Skin Health Dis. 2021 Dec 16;2(1):e82. doi: 10.1002/ski2.82 (PMC9060116; doi:10.1002/ski2.82)
Supplement: Supplementary file 1 — Supporting Information S1 [file SKI2-2-e82-s001.docx]

**A Preliminary Study of Peripheral T-cell Subsets in Porokeratosis Patients with *MVK* or *MVD* variants**

**Additional information**

**Index**

**Figure S1** Schematic of a novel mutation c.118_226+1337dup in *MVK* by CNVplex assay.

**Figure S2** Sanger sequencing chromatograms of NCs and PK patients at 5 novel mutation sites in *MVK* and *MVD.*

**Figure S3** Peripheral Vδ1 T cells remained unchanged in 22 PK patients compared with NCs.

**Figure S1. Schematic of a mutation c.118_226+1337dup in *MVK* by CNVplex assay.**


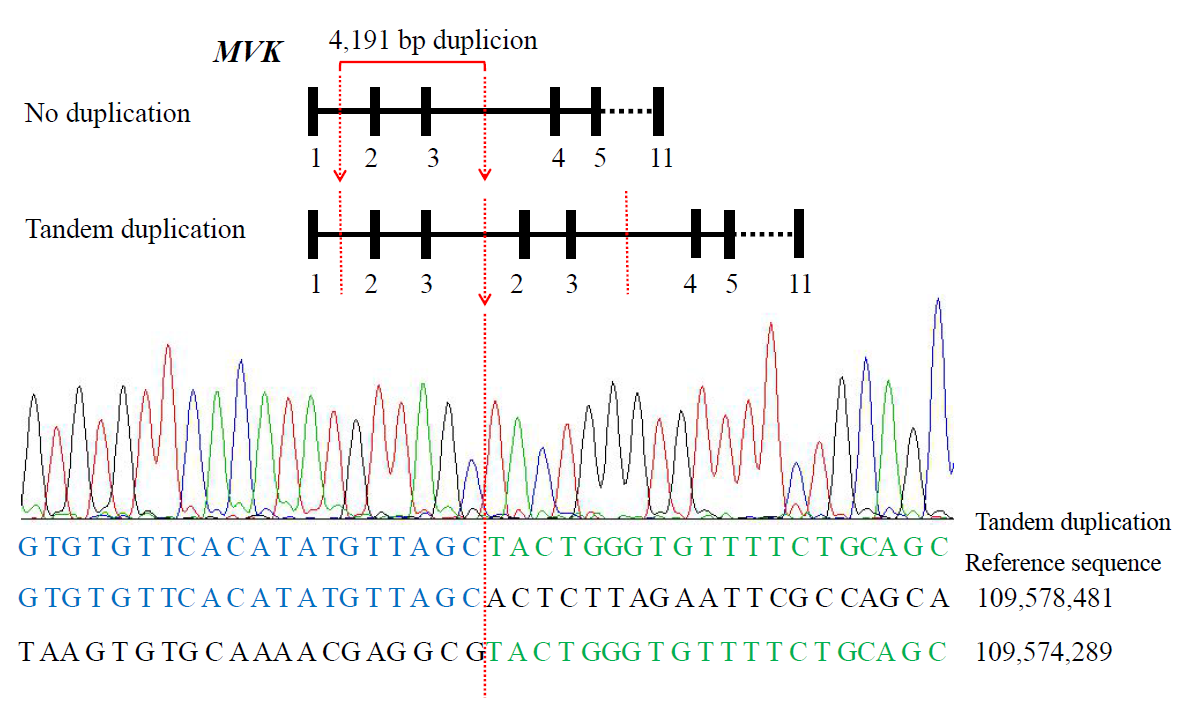


**Figure S2. Sanger sequencing chromatograms of normal controls (NCs) and PK patients at 5 novel mutation sites in *MVK* and *MVD.***

| **No.** | **Genes** | **Mutations** | **Chromatograms** | | |
| --- | --- | --- | --- | --- | --- |
|  |  |  | **From NCs** | | **From PK Patients** |
| **1** | ***MVK*** | **c.388_392delGATATinsC** | 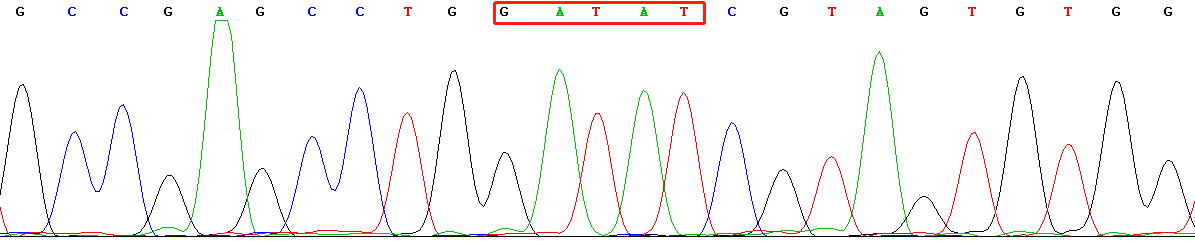 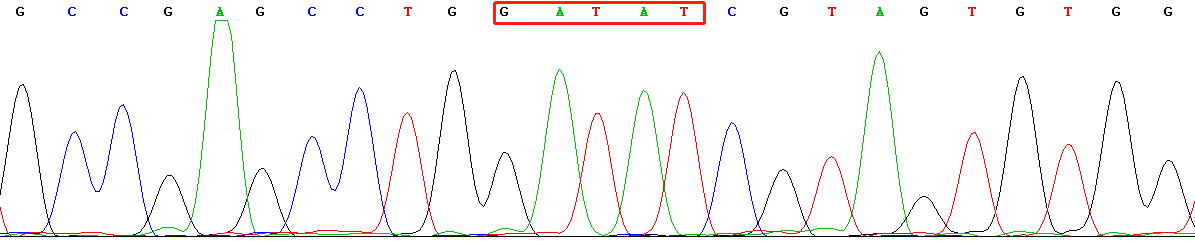 | 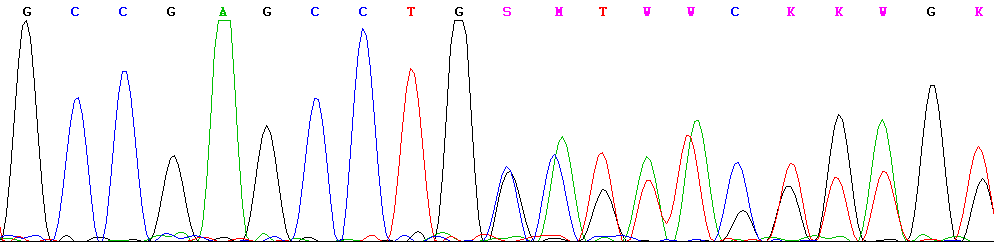 | |
| **2** | ***MVK*** | **c.613A>T** | 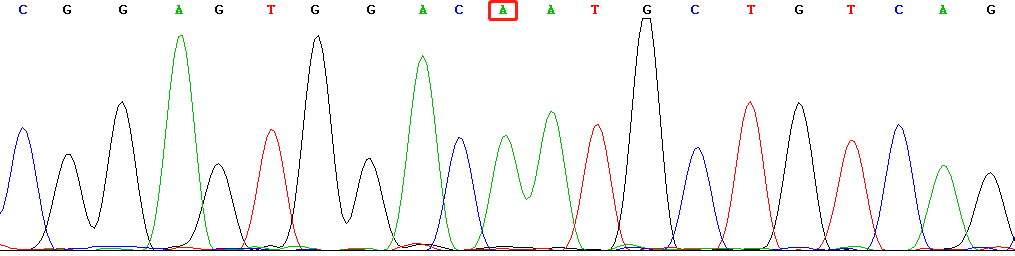 | 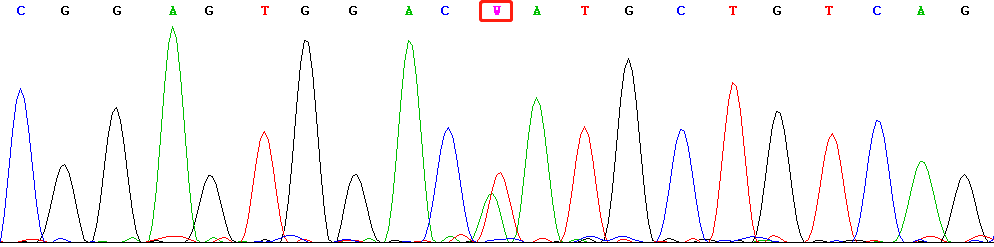 | |
| **3** | ***MVK*** | **c.768G>C** | 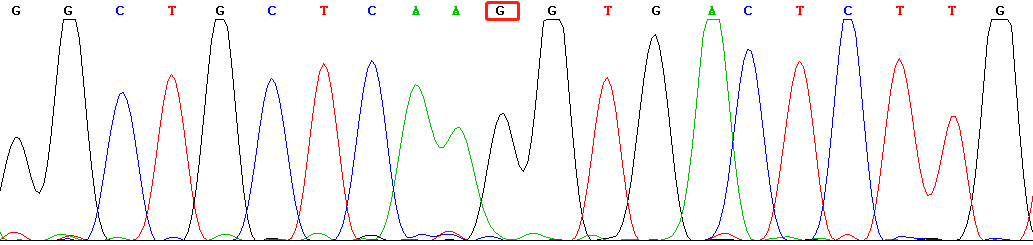 | 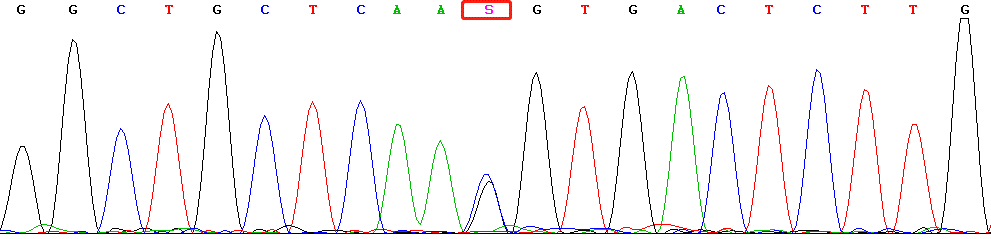 | |
| **4** | ***MVD*** | **c.250C>T** | 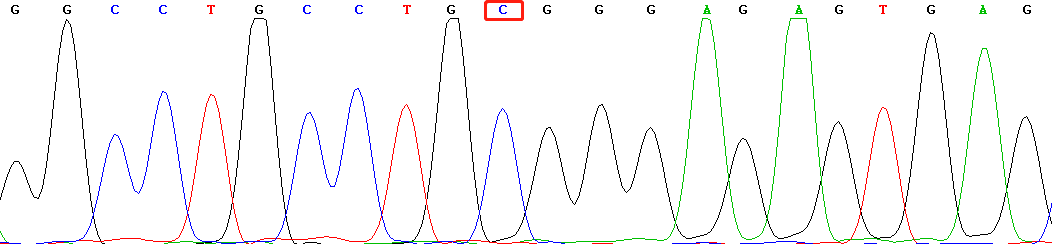 | 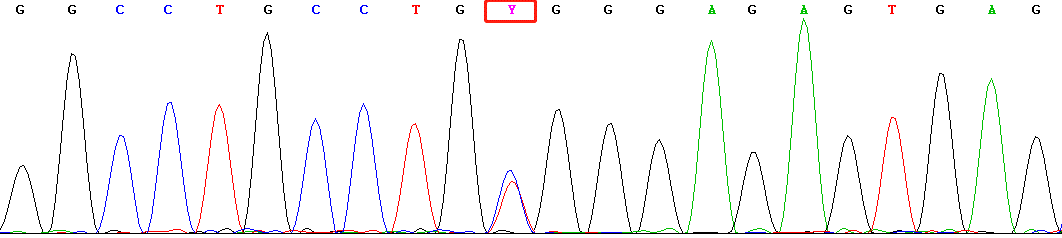 | |
| **5** | ***MVD*** | **c.988T>G** | 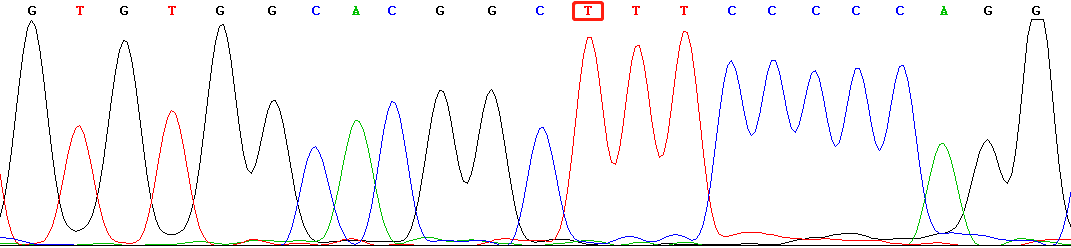 | 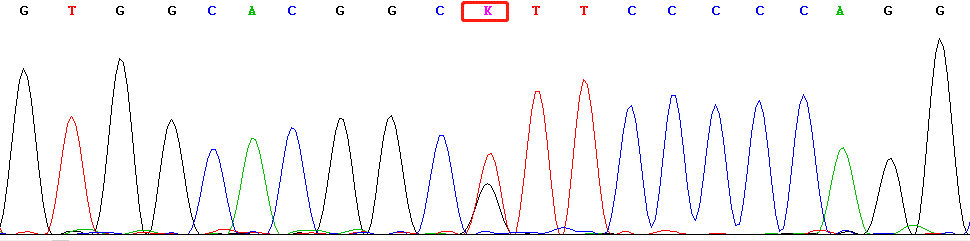 | |

**Figure S3. Peripheral Vδ1 T cells remained unchanged in 22 PK patients compared with NCs.** The representative flow cytometry data (a), and scatterplot graph (b) showing the frequencies of total Vδ1, Vδ1^+^Vγ9^+^ and Vδ1^+^Vγ9^−^ T cells in CD3^+^ T cells subsets.


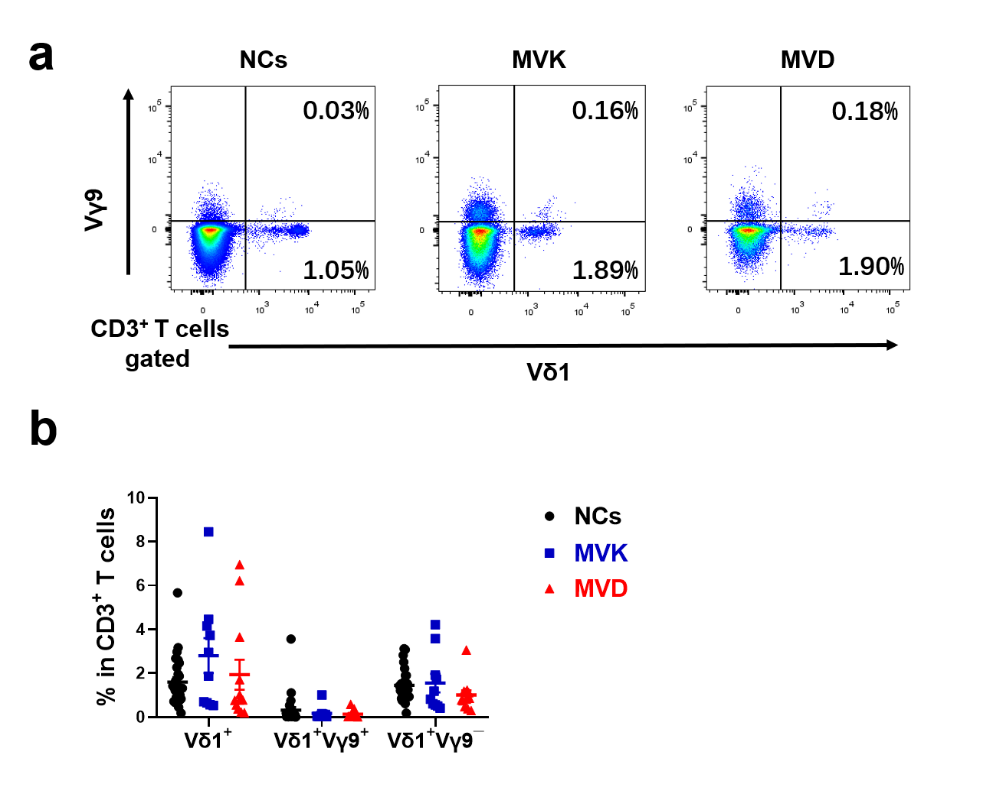


Notes: MVK: the PK patients with *MVK* variants (n = 10); MVD: PK patients with *MVD* variants (n = 12); NCs: normal controls (n = 27).
